# Deriving Treatment Decision Support From Dutch Electronic Health Records by Exploring the Applicability of a Precision Cohort–Based Procedure for Patients With Type 2 Diabetes Mellitus: Precision Cohort Study

**DOI:** 10.2196/51092

**Published:** 2024-05-01

**Authors:** Xavier Pinho, Willemijn Meijer, Albert de Graaf

**Affiliations:** 1 Netherlands Organisation for Applied Scientific Research (TNO) Utrecht Netherlands; 2 Nivel Nederlands Instituut voor Onderzoek van de Gezondheidszorg Utrecht Netherlands

**Keywords:** personalized care, electronic health records, EHRs, machine learning, type 2 diabetes mellitus, T2DM, decision-making

## Abstract

**Background:**

The rapidly increasing availability of medical data in electronic health records (EHRs) may contribute to the concept of learning health systems, allowing for better personalized care. Type 2 diabetes mellitus was chosen as the use case in this study.

**Objective:**

This study aims to explore the applicability of a recently developed patient similarity–based analytics approach based on EHRs as a candidate data analytical decision support tool.

**Methods:**

A previously published precision cohort analytics workflow was adapted for the Dutch primary care setting using EHR data from the Nivel Primary Care Database. The workflow consisted of extracting patient data from the Nivel Primary Care Database to retrospectively generate decision points for treatment change, training a similarity model, generating a precision cohort of the most similar patients, and analyzing treatment options. This analysis showed the treatment options that led to a better outcome for the precision cohort in terms of clinical readouts for glycemic control.

**Results:**

Data from 11,490 registered patients diagnosed with type 2 diabetes mellitus were extracted from the database. Treatment-specific filter cohorts of patient groups were generated, and the effect of past treatment choices in these cohorts was assessed separately for glycated hemoglobin and fasting glucose as clinical outcome variables. Precision cohorts were generated for several individual patients from the filter cohorts. Treatment options and outcome analyses were technically well feasible but in general had a lack of statistical power to demonstrate statistical significance for treatment options with better outcomes.

**Conclusions:**

The precision cohort analytics workflow was successfully adapted for the Dutch primary care setting, proving its potential for use as a learning health system component. Although the approach proved technically well feasible, data size limitations need to be overcome before application for clinical decision support becomes realistically possible.

## Introduction

The concept of learning health systems (LHSs) is an approach to health care that emphasizes continuous learning and improvement through the use of data and analytics [1]. Realizing that the US health care system was continuing to fall far short of its potential of delivering the best care at a lower cost, LHS was introduced in 2012 by the Institute of Medicine Committee on the Learning Health Care System in America as a “vision of what is possible if the nation applies the resources and tools at hand by marshaling science, information technology, incentives, and care culture to transform the effectiveness and efficiency of care - to produce high-quality health care that continuously learns to be better.” in their report *Best Care at Lower Cost: The Path to Continuously Learning Health Care in America* [2]. The underlying concept of the LHS is to harness the power of data and analytics to learn from every patient and feed the knowledge of *what works best* back to clinicians, public health professionals, patients, and other stakeholders to create rapid cycles of continuous improvement, which should allow to derive full benefits from leveraging data, systems, and human interconnectedness on an ever-increasing scale as is also seen in other sectors of the economy [3].

To help in deriving recommendations for implementation and evaluation criteria, LHSs have been conceptualized in frameworks from various perspectives, for example, impact on quality of health care [4], health care system evolution [5], and value creation [6].

The implementation of an LHS in practice requires the transition of a complex system of multiple stakeholders, processes, and technical (information) systems, irrespective of scale: local, regional, national, or international [7]. McDonald et al [8] recently identified the following as key enablers and actions required to enact LHSs: promotion of patient engagement, ensuring availability and access to data that are fit for purpose, keeping a focus on generating and implementing knowledge, creating organizational readiness, and stimulation of learning systems at different scales.

The diversity of factors identified illustrates the broadness of scope needed in assessing progress in the relatively novel field of LHSs. To date, very few reviews on the subject exist. Somerville et al [9] in a systematic review identified key implementation strategies, potential outcome measures, and components of functioning LHSs but stressed that further research is needed to better understand the impact of LHSs on patient, provider, and population outcomes and health system costs.

As is evident from this short overview of literature on LHSs, the use of data to create knowledge is one aspect of LHSs; however, it is a central one. Focusing specifically on the impact of the use of electronic health records (EHRs) on delivery or outcomes of health care, only 5 (12%) out of 43 eligible studies in a single available review study were found to document a medium-to-high level of evidence for impact [10]. This observation underlines the need for ongoing efforts to implement and evaluate the incorporation of EHR data analytics–driven knowledge generation in LHSs.

EHRs are electronic systems used to collect and store medical information of patients longitudinally over time and to collect and store information relevant to managing clinical workflows. As such, EHR data can be of a diverse nature. These data can be used for evaluation in various ways to extract knowledge [11]. Although traditionally done via statistical analyses, recent advances in machine learning techniques and applications have allowed the development and deployment of integrative algorithms that relate health care outcomes to multiple diverse sources of information present in EHRs. These algorithms can analyze large volumes of data to identify patterns and correlations and perform predictions. Approaches based on patient similarity are a typical recent example of this. A patient similarity approach tries to derive knowledge that is relevant to a given patient of interest who is presenting to the health care professional by analyzing information that is pertinent to clinically similar patients identified by a machine learning algorithm.

This study focuses on knowledge generation from EHRs in the Dutch primary health care setting based on a patient similarity approach. From a data analytics perspective, it seems appropriate to start exploring such an approach for a disease with substantial prevalence and incidence, that is, large volumes of data present in EHRs.

In 2013, approximately half of the Dutch population reported having at least 1 chronic disorder. One of the most common chronic disorders with a high disease burden is diabetes mellitus (DM). In 2021, approximately 4.9% of the Dutch population reported to have diabetes, of whom 90% (4.2%) had type 2 DM (T2DM) and the remaining had type 1 DM [12]. Therefore, T2DM was used as an example disease to explore the feasibility of an LHS approach in the Dutch primary care setting from a data analytical viewpoint.

In T2DM, a diminished insulin response in combination with insulin resistance results in hyperglycemia. Although T2DM is more common in participants aged >45 years, the numbers are increasing for younger individuals owing to a rise in obesity, sedentary lifestyle, and the intake of energy-dense diets [13]. Diabetes can be diagnosed and glycemic control can be monitored by measuring the glycated hemoglobin (HbA1c) levels or measuring the plasma glucose concentration. Diagnosis thresholds for HbA1c and plasma glucose concentration are >7% (53 mmol/mol) and >126 mg/dL (7 mmol/L), respectively [14]. Testing for HbA1c is convenient, fast, and standardized, but it is more costly and comes with a lower sensitivity than testing for plasma glucose. As a result, it has become a standard practice to use more frequent glucose measurements for regular monitoring and HbA1c measurements only at longer intervals or in special cases to assess the disease state and judge the necessity for treatment change. The initial steps in treating and managing T2DM involve lifestyle modifications, such as adopting a healthy diet, engaging in regular exercise, and quitting smoking. In the second step, when lifestyle modifications fail to achieve an adequate glycemic level, an antidiabetic medication is administered following national care standards. In the Netherlands, the first line of medication is metformin for non–high-risk patients and sodium-glucose transport protein 2 (SGLT-2) inhibitors for high-risk patients. There are various follow-up therapies, such as sulfonylureas, dipeptidyl peptidase 4 inhibitors, glucagon-like peptide-1 receptor agonists, SGLT-2 inhibitors, α-glucosidase inhibitors, and insulin [15].

In the Netherlands, general practitioners (GPs) are often the first point of contact for patients diagnosed with T2DM and act as gatekeepers to secondary care. Therefore, they play an important role in the diagnosis and treatment of these patients. To make treatment choices for individual patients with diabetes, physicians consider treatment guidelines and their own knowledge and experience, also—implicitly or explicitly—considering the patient’s perspective. Following the LHS approach, the decision-making may be supported by the on-demand availability of more objective information based on larger groups of comparable patients, such that the physician can see the actual data on improvements obtained after changing treatment in similar patients. This requires integration and sharing of data between physicians and health institutes. However, in practice, in Dutch primary health care settings, medical data sharing is still mostly limited to local or small regional settings, thus hampering the implementation of an LHS. The Netherlands Institute for Health Services Research (Nivel) hosts the Nivel Primary Care Database (PCD), in which routinely recorded data from EHRs from primary health care providers are collected and used to monitor health and use of health services in a representative sample of the Dutch population. Therefore, the Nivel-PCD was an appropriate database to study data analytical aspects for an LHS approach for patients with T2DM in the Dutch primary care setting.

This study aims to explore the applicability of a recently developed precision cohort analytics approach based on EHRs [16] as a candidate data analytical decision support tool focusing on data analytical aspects.

## Methods

### Overview

Our approach resides in patient matching and uses the precision cohort analytics methods developed by Ng et al [16]. In brief, there are different studies focused on building and applying matching methods. There is also evidence that patient similarity–based modeling outperforms population-based predictive methods [17]. Methods that can learn a disease-specific similarity metric by developing a locally supervised metric learning are very valuable to identify clinically similar patients. Similarity-based modeling algorithms can make use of different sources of data formats: textual data, numerical measurements, recorded signals, images, and vital signs. The algorithms used commonly are neighborhood-based algorithms, distance-based similarity metrics, correlation-based similarity metrics, cosine-similarity metrics, and cluster-based algorithms [17]. A clinical decision support (CDS) system that is intended to improve health care by improving medical decisions with clinical knowledge, patient information, and other health information intelligently filtered or presented at appropriate times [18] can for example include the following:

1. Mapping of clinical data from a patient to a specific point in a clinical pathway to advise treatment
2. Prediction of an individual’s responsiveness to different treatments based on the respective gene expression profiles
3. Identifying a patient as a candidate for a specific treatment based on a set of clinical characteristics with an associated desired treatment
4. Generating a patient trajectory graph from clinical data, capturing conditions, outcomes, interventions, and suggestions from medical guidelines at different patient group levels

The precision cohort treatment options approach used in this work is based on the abovementioned CDS approaches with the following adjustments:

1. Identification and extraction of relevant clinical treatment decision points (DPs) from the longitudinal patient data to use as events of interest for modeling and analysis
2. Selection and generation of features to determine patient similarity from different sources of information, such as guidelines, clinical measurements, prescriptions, consultations, and comorbidities
3. For a given patient of interest, creation of a precision cohort of patient events that are similar to the given clinical state
4. Demonstration of the available treatment options and statistics based on a retrospective analysis of the generated precision cohort

When a new patient presents with a need for a treatment decision, the approach can be used to create a precision cohort of the most clinically similar participants in the database and provide statistics on the past outcomes of different treatment decisions taken for these patients. This information can be used to support the health professional in their treatment choice. In this study, the published precision cohort analytics approach was adapted to the guidelines and EHR characteristics of the Dutch care setting. Results are compared with those of Ng et al [16], and their relevance for the Dutch setting is discussed. The outcomes may provide a further stimulus to ongoing initiatives to establish primary care medical data sharing at the national level in the Netherlands.

The major steps of this workflow are (1) EHR data extraction and preprocessing, (2) DPs identification and extraction (3), patient similarity model training, (4) precision cohort identification, and (5) treatment options and outcomes analysis.

### EHR Data Extraction and Preprocessing

The data were extracted from the Nivel-PCD [19]. Using an algorithm developed in prior research within Nivel-PCD [20], a total of 11,490 registered patients were identified as diagnosed with T2DM between 2012 and 2014, having at least 6 months of prior history in the database. The follow-up period was limited to a period of 5 years. Patients with incomplete data in this 5-year period were also included. The identification of patients with morbidities was done using an algorithm developed by Nielen et al [21] to construct episodes of illness based on routinely recorded EHRs. For the data extraction, only GP practices that permitted to use the data for scientific research were selected. Patient ID and GP practice ID are unique for these data and cannot be linked to other data sets to reduce the risk of tracking individual patients. For the selected 11,490 patients with T2DM, several tables were provided containing clinical information on 625,641 prescriptions (date, International Classification of Primary Care [ICPC] code, and ICPC description); 402,602 consultations (date and [Dutch] CTG code); 3,360,555 measurements (date, [Dutch GP association] Netherlands Huisartsen Genootschap code, and result or value); and 228,810 comorbidities (ICPC code and start and end date [based on the episodes of illness construct mentioned above] and type of comorbidity episode: 4 weeks, 8 weeks, 16 weeks, long-lasting, or chronic [21]).

To allow for a more explicit interpretation and analysis of the data, all clinical codes were replaced by the respective descriptions (Textbox 1), and dates were rewritten to a standard format, across all the tables.

**List of clinical codes and source of descriptions used.**

**Clinical measurement**

- Netherlands Huisartsen Genootschap [22]

**Prescription**

- Anatomical Therapeutic Chemical Classification System [23]

**Comorbidity code type**

- International Classification of Primary Care (International Classification of Primary Care codes were processed using a table provided by Nivel)

**Consultation code type**

- CTG [24]

### Decision Points

#### Identification and Extraction

From the several tables, points in time were extracted to serve as events of interest for the analysis and the modeling processes. These points are named DPs, and they are defined as points in time from the longitudinal data, of each patient, after the diagnosis date, where the disease is considered as being not under control either because HbA1c>7% or fasting glucose>7 mmol/L. These DPs thus represent opportunities to initiate a change in the treatment plan. This point must have another matching HbA1c or fasting glucose test in the follow-up period that classifies the outcome of the treatment decision, either as not under control (an HbA1c test>7% or a fasting glucose test>7 mmol/L) or as under control (an HbA1c test<7% or a fasting glucose test<7 mmol/L). Over time during longitudinal follow-up, a patient can have multiple DPs as long as the abovementioned criteria are fulfilled. A DP is composed of the following:

1. An index date:
  Date of an HbA1c or fasting glucose test that indicates a disease-uncontrolled situation along with the new treatment decision taken at this point.
2. A baseline period preceding the DP index date that represents the disease condition and the active treatment status, featuring the following:
  The treatment that was in effect
  The applicable clinical guidelines
  Data characterizing the condition of the patient
  All available clinical history until the DP index date. In case of multiple information available for the same field, the most recent was taken.
3. An observation period that follows the DP date, featuring the following:
  The new treatment
  The treatment outcome: either disease controlled or disease uncontrolled, during a period of 90 to 365 days after the index date

#### Target Outcome Variables

As described in the *Introduction* section, alternatively to using HbA1c International Federation of Clinical Chemistry and Laboratory Medicine as a target outcome variable, in this study, we also used fasting glucose, venous (laboratory) as a target outcome variable following the same procedure to extract DPs, but with a threshold of 7 mmol/L (Table 1). As mentioned in the *Introduction* section, these 2 clinical measurements are the most widely used tests for diagnosing T2DM. Although we have no information for both these metrics for all the patients, having 2 possible outcome judgment options allowed us to make wider use of the Nivel-PCD data available for each participant.

**Table 1:** Percentage of patients with measurements and those without and total number of decision points (DPs) that are controlled and uncontrolled for both glycated hemoglobin (HbA1c) and fasting glucose as target outcome variables (n=11,490).

			Target outcome variable		
			HbA1c		Fasting glucose
Patients with measurements, n (%)			10,375 (90.3)		10,824 (94.2)
**Number of DPs with outcomes**					
	Controlled	9,683		8,522	
	Uncontrolled	7,645		32,492	
Patients without any measurements, n (%)			1115 (9.7)		666 (5.8)

#### Treatments Considered

Many different prescriptions occur in the Nivel-PCD data set. To reduce the complexity of the analysis, we considered only medication-based treatments that specifically targeted T2DM (pharmacologic treatments), and we merged 3 different forms of healthy lifestyle advice encountered in the records (ie, “follow dietary advice,” “advice healthy food given,” and “advice physical activity given”) into a single nonpharmacological treatment, henceforth called “healthy lifestyle.” To select medications specifically targeting T2DM, treatment options present in the Nivel-PCD were compared against the Pharmaceutical Compass [25], containing independent pharmaceutical information for medical professionals, published by the Dutch National Healthcare Institute. Textbox 2 shows the resulting list of individual medications targeting DM that were considered in this study. Treatment options that combine multiple medications were also considered, for example, metformin and gliclazide (denoted as metformin_gliclazide).

As the dosage information was not available in the data sample used for this study, changes in drug dose were not captured and were interpreted as “no change” treatments.

For each DP, we assigned an active treatment (baseline period) and a new treatment (observation period). For assessing the active treatment of each DP, the medical history of the patient’s measurements was queried, and we applied the following reasoning: if neither pharmacologic nor lifestyle advice was found, then the DP was given a “no treatment” type of active treatment; if both pharmacologic and nonpharmacologic treatments were available, the 2 were merged (eg, metformin+healthy lifestyle). For assessing the new treatment, the same reasoning was applied; however, if the new treatment was the same as the active treatment, it was denoted as “no change.”

**List of medications targeting type 2 diabetes mellitus considered in this study.**

**Treatment options**

- Metformin
- Sitagliptin
- Insulin aspart
- Insulin degludec
- Insulin detemir
- Insulin glargine
- Insulin (human)
- Repaglinide
- Glimepiride
- Tolbutamide
- Gliclazide

#### Guidelines

Treatment decisions were made by the physicians based on clinical guidelines together with their personal past experience and considering the history and condition of the patient. The aim of using the guidelines is to improve the appropriateness of medical practice by leading to a better patient outcome while reducing costs, to aid authorities in deciding on the approval of drugs and devices, and to identify areas that need further research [26]. The guidelines for T2DM, published by the Dutch College of General Practitioners, including recommendations for the diagnosis, treatment, and management of patients, were incorporated in this study [27]. The relevant criteria for this study were derived from the guidelines to recommend medications and confirmed in a discussion with a GP:

1. Aged >70 years
2. Disease duration >10 years
3. BMI <25 kg/m^2^

#### Patient Condition

The clinical condition of patients was assessed using clinical measurements (which included data on, eg, BMI, diastolic blood pressure, and low-density lipoprotein), comorbidities, and consultation codes (Textbox 1). In addition, 2 patient condition criteria (mobility and mental state) were added based on the discussion with a GP, who emphasized that these are crucial aspects to consider when deciding which treatment is most appropriate.

Patients with mobility limitations are unlikely to perform physical exercise; therefore, even adhering to a restrictive healthy diet may not sufficiently control diabetes, necessitating medication sooner compared to patients without impaired mobility.

To assess the mobility state of the patient, we looked at comorbidities and measurements that indicated any possible obstacle to the ability to move. The list of comorbidities and measurements considered is represented in Table 2. We discriminated between the chronic and temporary duration of each comorbidity as used in the episodes of illness construct [21]. It has to be emphasized that the operationalization of reduced patient mobility as evident from Table 2 is a highly subjective choice made by the authors based on their intuitive understanding. It serves only for initial exploration of the feasibility of introducing additional GP considerations beyond the standard clinical guidelines.

Similarly, the mental state of the patient is also very important when deciding the appropriate treatment option. Patients who are going through events that alter their emotional state, affecting their concentration, positiveness, or willingness to adhere to treatment, may need a stricter treatment regime.

To assess the emotional state of the patient, we made an equally subjective choice of the different comorbidities that might be affecting their emotional state (Table 3).

**Table 2:** List of comorbidity and measurement types considered to assess the mobility state of patients. The upper part of the table shows the comorbidity International Classification of Primary Care (ICPC) code, description, and type of duration. At the bottom, it shows additional variables that were considered relevant to reflect mobility impairments, that is, the measurement type, Netherlands Huisartsen Genootschap (NHG) code description, and the duration.

Code			Code description		Duration
**ICPC**					
	L15	Knee symptoms or complaints		Temporary	
	L95	Osteoporosis		Chronic	
	L90	Osteoarthritis of the knee		Chronic	
	N17	Vertigo or dizziness (excluding H82)		Chronic	
	L14	Leg or thigh symptoms or complaints		Chronic	
	K90	Stroke or cerebrovascular accident		Chronic	
	L73	Fracture: tibia or fibula		Temporary	
	L03	Low back symptoms or complaints without radiation (excluding L86)		Chronic	
	L89	Osteoarthritis of the hip		Chronic	
	L76	Fracture: other		Temporary	
	R96	Asthma		Chronic	
	L79	Sprain or strain of other joints		Temporary	
	L74	Fracture: hand or foot bone		Temporary	
	L16	Ankle symptoms or complaints		Chronic	
	L97	Chronic internal knee derangement		Chronic	
	N18	Paralysis or weakness (excluding A04)		Chronic	
	L84	Osteoarthritis of the spine		Chronic	
	L77	Sprain or strain of the ankle		Temporary	
	L75	Fracture: femur		Temporary	
	L70	Infections musculoskeletal system		Temporary	
	L78	Sprain or strain of the knee		Temporary	
**NHG**					
	K93	Left foot amputation		Chronic	
	K94	Right foot amputation		Chronic	

**Table 3:** List of comorbidity types considered to assess the emotional state of patients. The table shows the comorbidity International Classification of Primary Care (ICPC) code, description, and type of duration.

ICPC code	Description	Duration
P01	Feeling anxious, nervous, tense, or inadequate	Temporary
P02	Acute stress or transient situational disturbance	Temporary
P20	Disturbances of memory, concentration, or orientation	Chronic
P74	Anxiety disorder or anxiety state	Chronic
Z18	Illness problem with a child	Temporary
Z19	Loss or death of a child	Chronic
P06	Disturbances of sleep or insomnia	Temporary
Z15	Loss or death of a partner	Temporary
A80	Accident or injury NOS^a^	Temporary
P03	Feeling depressed	Temporary
P99	Other mental or psychological disorder	Chronic
P72	Schizophrenia	Chronic
P76	Depressive disorder	Chronic
P73	Affective psychosis	Chronic
Z25	Problems resulting from assaults or harmful events	Temporary
P04	Feeling or behaving irritable or angry	Temporary
P77	Suicide attempt	Chronic
P98	Other or unspecified psychoses	Chronic
P70	Dementia (including senile and Alzheimer)	Chronic

^a^NOS: not otherwise specified.

### Patient Similarity Modeling

#### Feature Selection

Patient similarity was evaluated firstly based on the characteristics captured with the DP, that is, active treatment, applicable guidelines, and patient condition (including clinical history). In addition, to enrich the patient’s clinical information, more features were engineered. For instance, a Boolean feature named *has_chronic_comorbidity* was created to represent if the patient had or did not have a chronic comorbidity recorded at the time of interest in the medical history. In addition, the number of prescriptions, number of clinical measurements, number of consultations, and number of comorbidities from the disease diagnosis date until the DP date were added as candidates to the similarity features set.

As some algorithms cannot work directly with categorical data, one-hot encoding was applied to the variable *new_treatment*, converting this single column into N new columns, with N being the total number of different treatments (including combinations) observed in the data set. The same was done for the *active_treatment* variable.

The final data frame of DPs extracted and processed from the Nivel data set was composed of multiple feature types: identifiers, dates, and categorical and numerical data.

The resulting DPs data frame had a large amount of missing data. As a first selection step, only features that had at least 80% nonmissing values were retained [28].

Thereafter, the remaining missing values were imputed using the k-nearest neighbors (KNN)–based KNNImputer method from the *scikit-learn* library [29], with the following parameters: 2 nearest neighbors and a uniform weighting of all points in the neighborhood. The result of this step was a data frame of DPs without any missing values.

A further selection of the most salient features associated with disease control was done using the approach by Ng et al [16]. A total of 200 different L1-regularized logistic regression models (also known as the least absolute shrinkage and selection operator [LASSO] models) for predicting disease outcomes were created. LASSO is a linear regression technique that incorporates a penalty term to the sum of squared errors to shrink the coefficients toward 0 and perform feature selection. The penalty term is determined by the α parameter. Each model used a randomly selected subset (75%) of the data. The features selected by at least 150 of the 200 models were considered stable, and the remaining features were discarded. To find the best α parameter for the LASSO model, a grid search approach was applied. Different values of α were tested (100, 20, 10, 2, 1.67, 1.43, 1.25, 1.11, and 1), and the value corresponding to the highest *F*_1_-score was selected.

As the 2 target outcome variables were unbalanced, we used the *F*_1_ score [30], rather than accuracy, as a metric of prediction performance.

It is important to note that 2 different data frames were built, and separate similarity models were built, for the 2 outcome variables HbA1c and fasting glucose. Each of the data frames was split into 2 sets: a training set that was used to train the patient similarity model and a scoring set used as a repository of clinical events and to create multiple precision cohorts.

#### Similarity Model Training

The similarity model used the set of stable features obtained from the previous steps to calculate patient similarity. This model learns a T2DM distance measure that is a modified version of the Mahalanobis distance (MD). The MD measures the distance relative to a centroid or central point, in which all means from all variables intersect; the larger the MD, the further away from the centroid the data point is. The MD can also be used to calculate the distance between 2 points, or in this case, 2 patients (x_i_ and x_j_) using the covariance matrix. Here, a modified version of the MD formula was used, with the covariance matrix replaced by a weight matrix W, which is learned from the training data.



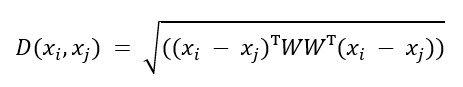



**(1)**

The similarity model sets the weight matrix to maximize the target class discriminability (disease control state) by adjusting the weights of every feature. This was done by locally separating points from different classes while keeping together points that belong to the same class, using a large margin nearest neighbor (LMNN) [31] machine learning algorithm.

The number of points to consider for the calculations is defined by the number of neighbors parameter (K). Different values of K were tested: 2, 3, 4, 5, 6, and 7.

To assess the effect of the LMNN algorithm, we compared the performance of a KNN classification model [29] on the raw data and the data transformed by the similarity model [32]. For the KNN algorithm, a grid search approach was used to find the best set of parameters:

1. Number of neighbors (N): (3, 4, 5, ..., or 30)
2. Weight function: uniform (all points in each neighborhood weighted equally) or distance (closer neighbors of a query point will have a greater influence than neighbors that are further away).

The learned similarity weights for each variable for T2DM separately for HbA1c and fasting glucose as target outcome variables are shown in Multimedia Appendices 1 and 2.

### Precision Cohort Construction

Precision cohort construction consists of selecting the most clinically similar patient DPs based on the characteristics of the patient of interest at the time of the consultation. The process of generating precision cohorts needs to ensure that the baseline confounders are adjusted so that the effect analysis is valid (ie, a good covariate balance is achieved). The selection was done in a 2-step procedure: a filtering step and a similarity rating.

To filter the patients who are more similar to the patient of interest, filter variables were generated. These filter variables were composed of guidelines plus the active treatment. For instance, if the patient was aged 80 years and was currently taking metformin, the filter variable was age_above_70y+metformin. By using this filter variable, it was ensured that only patients aged >70 years and who were taking metformin were selected for the precision cohort. It is important to mention that only data from the “baseline period” of the DP were used for the filtering process, which means that the treatment decision at the time of the DP (index date) was not considered, as we want to analyze the entire pool of new treatments in the precision cohort. Combining the set of guidelines with the set of active treatments resulted in a very large number of filter cohorts of widely varying sizes. Considering that it is difficult to recommend treatment options for patients with clinically odd profiles as this method requires a large patient pool to recommend statistically significant treatment options, we focused only on the most representative cohorts, that is, those with >200 DPs.

For the second step (similarity rating), the similarity model explained in the *Similarity Modeling* section was used to calculate the similarity scores for the filtered DPs. The similarity score is a distance metric; thus, smaller scores indicate a higher similarity between a patient’s DP and the DP of the patient of interest. The similarity score was converted to a normalized distance using a minimum-maximum normalization method to allow an easier interpretation of these scores.

Thereafter, the final precision cohort was generated by retaining only the “most similar” patients. However, reducing the cohort size may compromise the cofactor balance. Therefore, the covariate balance of the precision cohort with varying sizes was calculated to assess bias and matching validity by comparing the “no treatment change” (new treatment is the same as the active treatment) with the “treatment change” (new treatment different from the active treatment) groups. Covariate balance was calculated as the difference in the means of each covariate between the 2 groups divided by the SD of the treated group [33]. The closer this value was to 0, the better balance we had between the groups.

To have a trade-off between the covariate balance value and the number of DPs in the precision cohort, a normalized distance value of 2 was defined as a cutoff. This value was chosen after visual inspection of several covariate balance plots for different precision cohorts. Some studies agreed that covariate balance values <0.1 were satisfactory [34], although another study suggested that a value of 0.25 was good enough [35]. With a normalized distance cutoff value of 2, a covariate balance of 0.1 was achieved for most of the precision cohorts.

In summary, the precision cohort construction process involved the following:

1. Filtering DPs with the same filter variables as the patient of interest
2. Calculating the similarity scores for the filtered DPs
3. Ranking DPs based on similarity scores (normalized distance)
4. Retaining only the DPs with a normalized distance <2

### Treatment Options and Outcomes Analysis

Treatment outcomes analyses were performed both from a global perspective (ie, across all filter cohorts) and a personalized perspective (ie, in the precision cohorts). The latter can be used as a retrospective analysis to generate personalized treatment options for a given patient of interest.

The set of DPs in the precision cohort was grouped by treatment decision. For each of these treatment groups, we computed the following:

1. The number of DPs
2. The percentage of DPs that have a controlled outcome
3. The difference in the respective outcome compared with the “no treatment” change option
4. A statistical significance assessment using a Bonferroni corrected χ^2^*P* value of 0.5 to adjust for multiple comparisons

To better visualize the difference between the different treatments for each precision cohort, the results were presented in a Sankey diagram [36].

To reduce the number of treatment options with a low number of DPs, we decided to only include those with at least 1% of the total DPs in the precision cohort, with a minimum of 10 DPs. For instance, if the precision cohort had 2000 DPs, we only included treatment options with at least 20 DPs.

### Ethical Considerations

This study was approved according to the governance code of Nivel-PCD (NZR-00320.048). The use of EHRs for research purposes is allowed under certain conditions. When these conditions are fulfilled, neither obtaining informed consent from patients nor approval by a medical ethics committee are obligatory for this type of observational study containing no directly identifiable data (Art. 24 General Data Protection Regulation Implementation Act jo art. 9.2 sub j General Data Protection Regulation).

## Results

Separate analyses were conducted for HbA1c and fasting glucose as target outcome variables.

### HbA1c Outcome Scenario

#### Decision Points

For the HbA1c scenario, we found 17,328 DPs across the available longitudinal data from the 11,490 patients with T2DM.

Although seemingly large, this was still 10-fold lower compared with previous work by Ng et al [16], who retrieved >171,000 DPs for 24,373 patients with T2DM from their data set, for HbA1c as an outcome.

The processed HbA1c set was split into 5199 DPs for the training set and 12,129 DPs for the scoring set. As the class (disease control) was unbalanced, we had to ensure that we had the same proportion of each class for both training and scoring sets. For this case, of the 17,328 DPs, we had 9704 (56%) uncontrolled DPs and 7624 (44%) controlled DPs. This same proportion was maintained for the training set: there were 2905 uncontrolled DPs and 2294 controlled DPs in the training set and 6793 uncontrolled DPs and 5336 controlled DPs in the scoring set.

#### Patient Similarity Modeling

The methods of feature generation, missing data imputation, and feature selection explained in the *Methods* section were applied to this subset. The LASSO model α value used was 1.43, with an *F*_1_-score of 0.613.

The training set was used to construct a similarity model. The optimal k value for the LMNN algorithm was 3. Both the raw data and the data transformed with the similarity model were subjected to the KNN algorithm. The most optimal parameter combination for the KNN algorithm was determined to be N=6 neighbors and a weight function based on distance (data not shown).

Next, the tuned version of the KNN algorithm was used to evaluate the LMNN algorithm performance (refer to the *Methods* section). The *F*_1_-score was 0.606 for raw data versus 0.613 for transformed data. Thus, indeed, the LMNN algorithm resulted in improved classification performance.

The learned similarity weights for each variable for HbA1c as the target outcome variable are shown in Multimedia Appendix 1. These weights are disease specific for T2DM. A total of 26 features were retained for the similarity model. Interestingly, there was rather limited variation in size: similarity weights were all of comparable value (typically ranging between 0.4 and 0.6), except for features “#comorbidities” and “Systolic BP.”

#### Precision Cohort Construction

**Figure 1:**
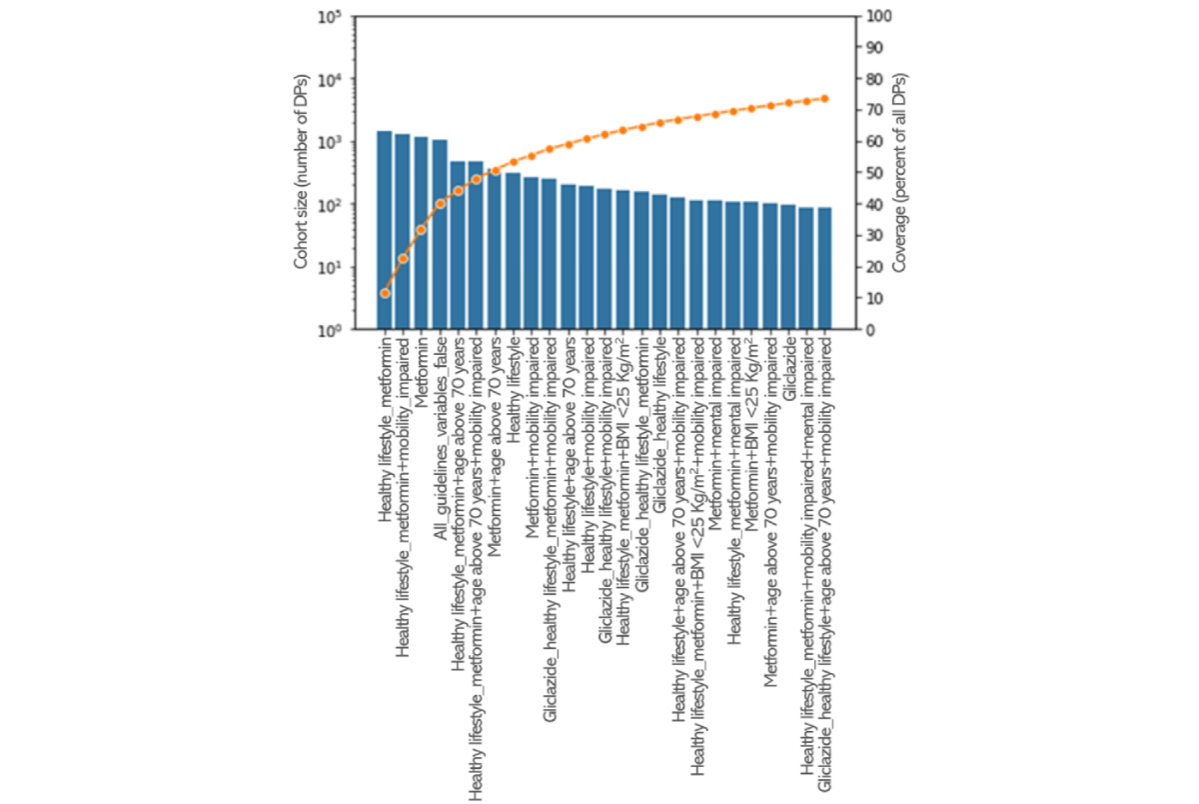
The 25 largest cohorts for glycated hemoglobin (HbA1c) as a target outcome variable based on the filter variables. The blue bars represent the number of decision points (DPs) on a logarithmic scale (left vertical axis), and the orange line shows the cumulative coverage on a linear percentage scale (right vertical axis).

The largest observed filter cohorts for HbA1c are shown in Figure 1. These 25 cohorts covered approximately 75% of all the DPs in the scoring set. Of these 25 cohorts, 20 (80%) had >100 DPs. Only 10 cohorts had >200 DPs, considered potentially useful for constructing precision cohorts. As expected, the cohorts containing metformin were the largest ones as it is the first line of medication for the treatment of T2DM. The cohort “healthy lifestyle_metformin” was the largest cohort for the HbA1c outcome scenario. The cohort “all_guidelines_variables_false” also contained a large number of DPs. In this cohort of patients, who were aged ≤70 years, were not mobility or mentally impaired, and had a BMI of ≥25 kg/m^2^, no antidiabetic treatment (medication or healthy lifestyle advice) was administered or the information was not registered. Furthermore, of the 25 largest cohorts, 17 (68%) had “healthy lifestyle,” 16 (64%) had “metformin,” 11 (44%) had “mobility impaired,” 7 (28%) had “age_above_70y,” and 3 (12%) had “mental impaired.” No other pharmacological treatments than metformin and gliclazide (6 occurrences) were represented in the 25 largest cohorts.

**Figure 2:**
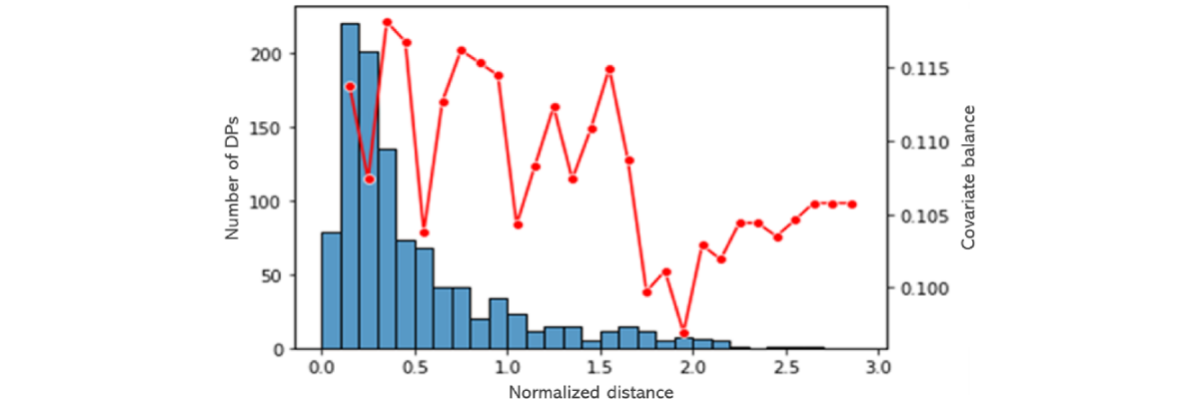
Illustration of the metformin precision cohort generation for the glycated hemoglobin (HbA1c) outcome target. The decision points (DPs) in the subset were grouped by normalized distance (similarity score) to the patient of interest. The blue bars represent the number of DPs per bar or grouped DPs (left vertical axis), and the red dotted line shows the cumulative covariate balance values for the different bars or grouped DPs (right vertical axis).

Following the procedures explained in the *Precision Cohort Construction* section, precision cohorts were generated for a number of randomly chosen patients from various filter cohorts. Figure 2 shows a covariate balance plot for 1 randomly chosen patient in the metformin filter cohort as an example. As can be appreciated from the figure, the best cofactor balance was achieved for normalized distance 2.0.

#### Treatment Options and Outcomes Analysis

**Figure 3:**
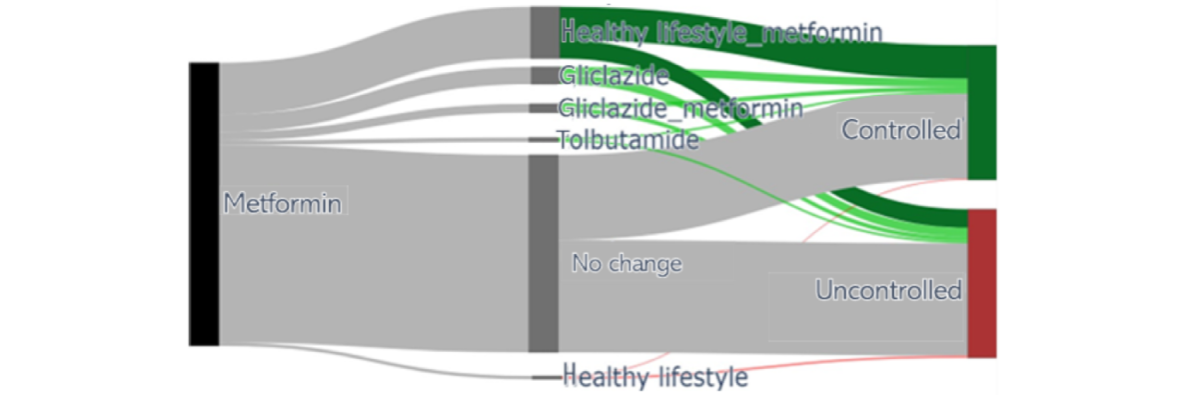
Personalized treatment options observed in the precision cohort for a given patient belonging to the filter cohort “Metformin,” using glycated hemoglobin (HbA1c) as the target outcome variable. The initial node, in black, includes all the DPs in the precision cohort for the particular patient of interest. This patient has all the guidelines variables as “False” and is currently on a metformin prescription. Each pathway from the initial node is a different treatment decision observed in the data. The thickness of the pathway is proportional to the number of DPs, and it is assigned with a label that represents the new medication, and the percentage of DPs controlled in the follow-up. The “no change” treatment option is colored in gray, and it is considered the baseline treatment option. The terminal nodes represent the outcome; the nodes in green denote the DPs that achieved control, whereas those in red indicate the uncontrolled ones. Treatment options with better control than the baseline option are colored in green, whereas those with a worse control are colored in red. Treatment options with a statistical significance are colored dark green or dark red.

Table 4 shows the overall percent controlled of the 20 most representative treatment options on a global scale, that is, across the cohorts based on the filter variables. In >47% of cases, the GP decided to continue the current treatment despite the HbA1c levels being classified as uncontrolled. Despite no change, >44% of patients had their HbA1c levels subsequently “controlled” during the follow-up. Some alternatives, however, had much better outcomes. For instance, the option of stopping the “no treatment” option and starting taking metformin, that is, the first line of medication treatment according to the guidelines, resulted in 57.6% (213/370) of DPs with HbA1c controlled, whereas starting treatment with healthy lifestyle advice resulted in >64% (63/98) controlled outcomes. Despite this difference, starting treatment with metformin was chosen almost 4 times more often than starting with healthy lifestyle treatment (370 vs 98 cases). The combination of metformin and lifestyle advice was beneficial over continuing each of these treatments as a single treatment. This list is relevant for analyzing the global picture of the available treatment options and the corresponding outcomes. However, for individual patients, the treatment option analysis might differ from the overall picture depending on the precision cohort that more closely reflects the clinical scenario for the particular patient. As an example of such a case-specific analysis, Figure 3 uses a Sankey diagram to represent the different treatment options for a given patient that belongs to the cohort “Metformin.”

In Table 4, it can be noticed that adding healthy lifestyle advice to the metformin prescription was a treatment option with a statistically significant better-associated outcome (210/324, 64.8%), whereas changing from metformin to only healthy lifestyle advice alone led to a worse disease outcome in the precision cohort for this particular patient. Changing the treatment to gliclazide or tolbutamide, or adding gliclazide to metformin, also resulted in better outcomes; however, the differences were not statistically significant because of the low number of cases involved. We can also see that from the most similar patients who kept taking only metformin, 45.69% (605/1324) of the patients improved their disease condition. For the remaining 54.31% (719/1324) of the patients, the outcome was “uncontrolled” in the follow-up.

**Table 4:** The 20 largest observed global treatment option groups for glycated hemoglobin as the target outcome variable. The list shows the size of each group and the associated percentage controlled during follow-up (n=12,129).

Treatment option	Frequency, n (%)	DPs^a^ controlled, n (%)
No change	5747 (47.38)	2541 (44.21)
Metformin_new+healthy lifestyle_metformin_stop^b^	1324 (10.92)	605 (45.69)
Healthy lifestyle_new+healthy lifestyle_metformin_stop^c^	402 (3.31)	170 (42.29)
Metformin_new+no treatment_stop^b^	370 (3.05)	213 (57.57)
Healthy lifestyle_metformin_new+metformin_stop^b^	324 (2.67)	210 (64.81)
Healthy lifestyle_metformin_new+healthy lifestyle_stop^b^	246 (2.03)	165 (67.07)
Healthy lifestyle_metformin_new+no treatment_stop^b^	222 (1.83)	138 (62.16)
Gliclazide_healthy lifestyle_new+healthy lifestyle_metformin_STOP^c^	217 (1.79)	86 (39.63)
Metformin_new+healthy lifestyle_STOP^b^	169 (1.39)	91 (53.85)
Gliclazide_new+healthy lifestyle_metformin_stop^c^	151 (1.24)	65 (43.05)
Gliclazide_new+metformin_stop^b^	148 (1.22)	80 (54.05)
Gliclazide_metformin_new+gliclazide_healthy lifestyle_metformin_stop^c^	147 (1.21)	38 (25.85)
Gliclazide_new+gliclazide_healthy lifestyle_stop^c^	123 (1.01)	41 (33.33)
Healthy lifestyle_new+no treatment_stop^b^	98 (0.81)	63 (64.29)
Gliclazide_healthy lifestyle_metformin_new+healthy lifestyle_metformin_stop^b^	68 (0.56)	32 (47.06)
Gliclazide_metformin_new+metformin_stop^c^	59 (0.49)	30 (50.85)
Metformin_new+gliclazide_healthy lifestyle_stop^b^	59 (0.49)	16 (27.12)
Gliclazide_healthy lifestyle_new+gliclazide_healthy lifestyle_metformin_stop^c^	58 (0.48)	17 (29.31)
Healthy lifestyle_metformin_new+gliclazide_healthy lifestyle_stop^c^	58 (0.48)	12 (20.69)
Healthy lifestyle_metformin_new+gliclazide_healthy lifestyle_metformin_stop^c^	57 (0.47)	21 (36.84)

^a^DP: decision point.

^b^Treatment options with a controlled percentage higher than the “no change” treatment option.

^c^Treatment options with a lower percentage than the “no change” treatment option.

### Fasting Glucose Scenario

#### Decision Points

For the fasting glucose target outcome variable, we found 41,014 DPs across the available longitudinal data, that is, approximately 2.5 times more than for the HbA1c scenario.

The processed data set was split into 12,304 DPs for the training set and 28,710 for the scoring set. Again, the disease control target outcome variable was not balanced; thus, the proportions were kept the same for both training and scoring sets. In this case, we had 79% (32,401/41,014) uncontrolled DPs and 21% (8613/41,014) controlled DPs. Accordingly, the same proportion was kept for both the training and the testing sets.

#### Patient Similarity Modeling

Similarly to the HbA1c scenario, the methods of feature generation, missing data imputation, and feature selection explained in the Methods section were applied for the fasting glucose scenario. The LASSO model α value used was 1.67, with an *F*_1_-score of 0.691. The training set was used to train the similarity model. The optimal k value for the LMNN algorithm was 5. The KNN algorithm was applied to the raw data and the transformed data. The best combination of parameters was N=6 and weight function=distance, that is, the same as that for the HbA1c scenario (data not shown).

Next, the tuned version of the KNN algorithm was used to evaluate the LMNN algorithm performance. The *F*_1_-score was 0.849 for raw data and 0.856 for transformed data. Thus, only a small improvement in classification performance was achieved by the LMNN algorithm.

The learned similarity weights for each variable for fasting glucose as target outcome variable are shown in Multimedia Appendix 2. These weights are disease specific for T2DM. A total of 48 features were retained for the similarity model, 22 more than that for HbA1c. In total, 25 features were of the “active _treatment_”-type, whereas for HbA1c, only 5 features were of this type. This indicates that the active treatment was much more predictive of disease outcome than in the HbA1c scenario. With approximately half of weight values between 0.4 and 0.65, and half between 0.7 and 1.15, there was much more variation in the size of weights compared with the HbA1c scenario. Similar to the HbA1c scenario, the feature “Systolic BP” had a low weight, probably because it had little discriminating power.

#### Precision Cohort Construction

**Figure 4:**
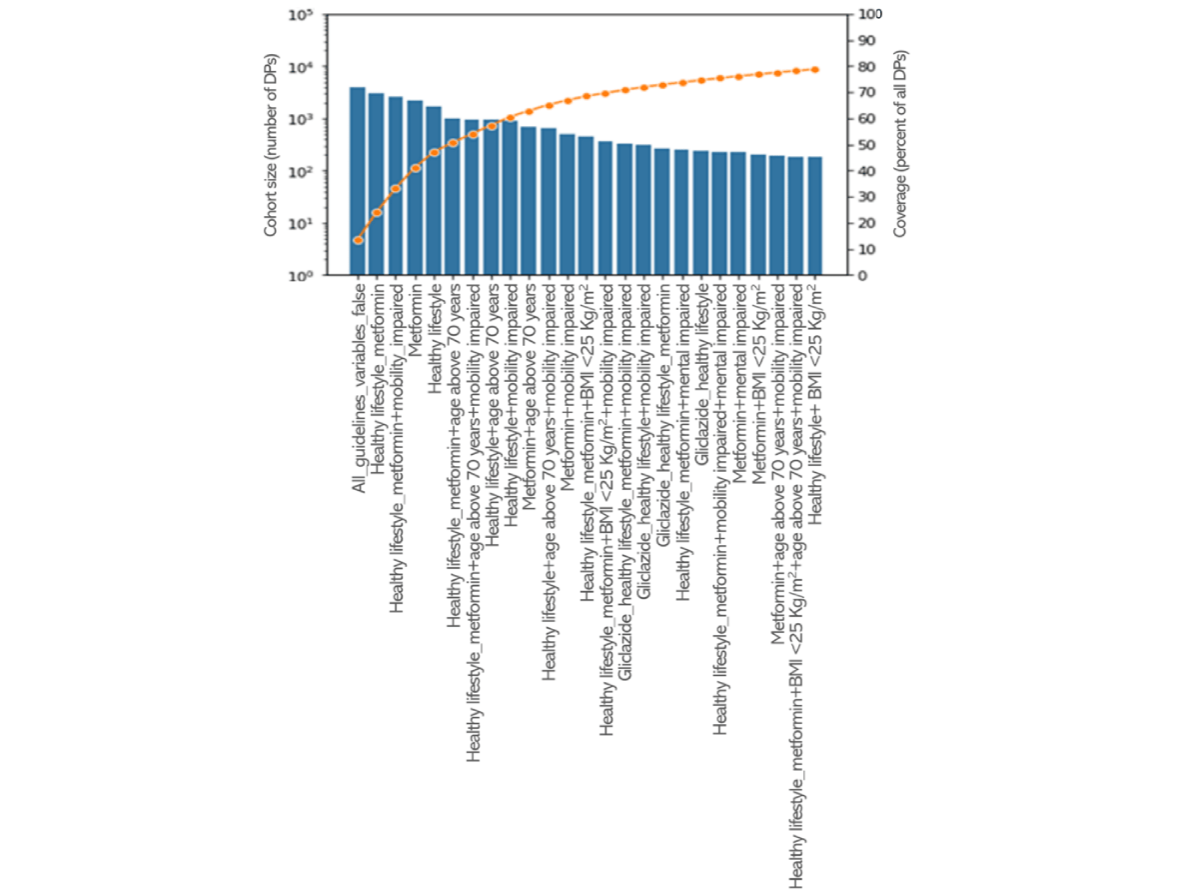
The 25 largest cohorts for fasting glucose as target outcome variable based on the filter variables. The blue bars represent the number of decision points (DPs) on a logarithmic scale (left vertical axis), and the orange line shows the cumulative coverage on a linear percentage scale (right vertical axis).

In Figure 4, the 25 largest observed filter cohorts for fasting glucose are represented. Together, these covered approximately 80% of DPs in the data set. In line with the much larger number of DPs compared with the HbA1c scenario, 20 of the largest filter cohorts had >200 DPs. Contrary to the HbA1c scenario, here the largest cohort was the “all_guidelines_variables_false,” followed by the “healthy lifestyle_metformin cohort,” which is in accordance with what we expected as the fasting glucose values are used more frequently to diagnose T2DM and most patients do not start medication immediately. Furthermore, of the 25 largest cohorts, 17 (68%) had “healthy lifestyle,” 16 (64%) had “metformin,” 11 (44%) had “mobility impaired,” 7 (28%) had “age_above_70y,” and 3 (12%) had “mental impaired,” all identical to the HbA1c scenario. Moreover, 23 (92%) of the 25 largest cohorts for the fasting glucose scenario were also in the list of the 25 largest cohorts for the HbA1c scenario, and the ranking in size was similar (≤3 positions difference). No other pharmacological treatments than metformin and gliclazide (4 occurrences) were represented in the 25 largest cohorts.

Various precision cohorts for randomly selected patients were constructed to verify that the chosen normalized distance threshold of 2.0 indeed resulted in optimal cofactor balance overall (data not shown) for the fasting glucose outcome scenario as well.

#### Treatment Options and Outcomes Analysis

Table 5 shows the overall controlled percentage of the 20 most representative treatment options, that is, across all filter cohorts. In >57.4% (16,480/28,709) of the cases, the existing treatment was continued, and in only 23.29% (3839/16,480) of the cases, this led to patients becoming “controlled” as judged by the fasting glucose value. Interestingly, in this scenario, the option of starting to take metformin and stopping the “no treatment” resulted in a lower success percentage when compared with the “no change” option. More differences are apparent when comparing with the HbA1c scenario; overall, the percentages of a “controlled” outcome seem more than 2-fold lower and never >37%, thereby seemingly indicating a much more pessimistic perspective.

**Figure 5:**
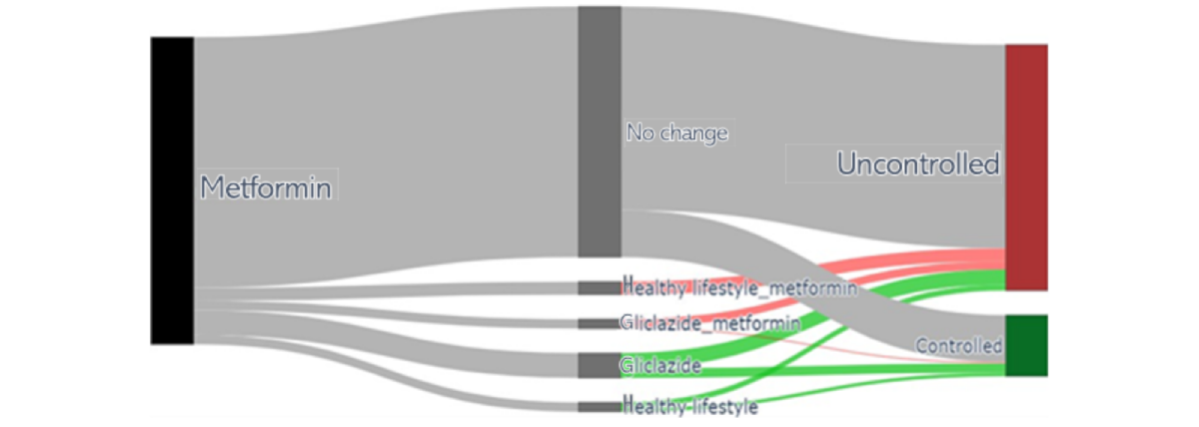
Personalized treatment options observed in a precision cohort for a given patient belonging to the filter cohort “Metformin with mobility impairments,” using fasting glucose as target outcome variable. The initial node, in black, includes all the decision points (DPs) in the precision cohort for the particular patient of interest. This patient has all the guidelines variables as “False,” except the mobility impairment as “True,” and is currently on a metformin prescription. Each pathway from the initial node is a different treatment decision observed in the data. The thickness of the pathway is proportional to the number of DPs, and it is assigned with a label that represents the new medication, and the percentage of DPs controlled in the follow-up. The “no change” treatment option is colored in gray, and it is considered the baseline treatment option. The terminal nodes represent the outcome; the nodes in green denote the DPs that achieved control, whereas those in red indicate the uncontrolled ones. Treatment options with better control than the baseline option are colored in green, whereas those with a worse control are colored in red. No treatment options with statistical significance were found in the precision cohort for this patient.

Figure 5 represents the different treatment options observed in a precision cohort for a given patient that belongs to the cohort “Metformin with mobility impairments” using a Sankey diagram.

Analyzing the Sankey diagram in Figure 5, we can notice that for patients with mobility impairments who were taking metformin, only a small proportion of those who were kept on the same prescription met with an improved disease control outcome. The ones who changed from metformin to gliclazide showed an improvement, although the number of DPs for these 2 options was not very large, and the result was not statistically significant as a consequence. These results showed that the set of patients, similar to the patient of interest, had difficulties in having the fasting glucose controlled and might need more attention from the health care professionals. Many other cohorts could be included; we chose 2 examples that had a decent number of patients to show how this approach works in practice.

**Table 5:** The 20 largest observed global treatment option groups for fasting glucose as the target outcome variable. The list shows the size of each group and the associated percentage controlled during follow-up (n=28,710).

Treatment option	Frequency, n (%)	DPs^a^ controlled, n (%)
No change	16,480 (57.4)	3839 (23.29)
Metformin_new+healthy lifestyle_metformin_stop^b^	3130 (10.9)	586 (18.72)
Healthy lifestyle_new+healthy lifestyle_metformin_stop^b^	1011 (3.52)	201 (19.88)
Metformin_new+no treatment_stop^b^	639 (2.23)	110 (17.21)
Healthy lifestyle_new+no treatment_stop^c^	638 (2.22)	203 (31.82)
Healthy lifestyle_metformin_new+metformin_stop^b^	612 (2.13)	59 (9.64)
Healthy lifestyle_metformin_new+healthy lifestyle_stop^c^	429 (1.49)	157 (36.6)
Healthy lifestyle_metformin_new+no treatment_stop^b^	399 (1.39)	69 (17.29)
Gliclazide_metformin_new+gliclazide_healthy lifestyle_metformin_stop^b^	284 (0.99)	28 (9.86)
Metformin_new+healthy lifestyle_stop^c^	265 (0.92)	97 (36.6)
Gliclazide_healthy lifestyle_new+healthy lifestyle_metformin_stop^b^	259 (0.9)	32 (12.36)
Gliclazide_new+gliclazide_healthy lifestyle_stop^b^	223 (0.78)	39 (17.49)
Gliclazide_new+healthy lifestyle_metformin_stop^c^	204 (0.71)	54 (26.47)
Gliclazide_new+metformin_stop^b^	165 (0.57)	22 (13.33)
Gliclazide_healthy lifestyle_metformin_new+healthy lifestyle_metformin_stop^b^	105 (0.37)	5 (4.76)
Healthy lifestyle_new+metformin_stop^b^	102 (0.36)	15 (14.71)
Metformin_new+gliclazide_healthy lifestyle_stop^b^	102 (0.36)	13 (12.75)
Gliclazide_metformin_new+gliclazide_healthy lifestyle_stop^b^	98 (0.34)	8 (8.16)
Healthy lifestyle_metformin_new+gliclazide_healthy lifestyle_stop^b^	93 (0.32)	6 (6.45)
Metformin_tolbutamide_new+healthy lifestyle_metformin_tolbutamide_stop^b^	91 (0.32)	2 (2.2)

^a^DP: decision point.

^b^Treatment options with a controlled percentage lower than the “no change” treatment option.

^c^Treatment options with a controlled percentage higher than the “no change” treatment option.

## Discussion

### Principal Findings

This contribution evaluated the feasibility of using a precision cohort treatment options approach to generate personalized treatment options for patients with T2DM in the Dutch primary care setting. The approach involves the identification of relevant clinical treatment DPs from the longitudinal patient data to use as events of interest for modeling and analysis, patient similarity modeling for precision cohort construction, and treatment options and outcomes analysis as main elements. All procedures functioned well from a technical viewpoint; however, data size limitations proved challenging for reaching statistical significance for differences in outcomes of multiple treatment options.

### Decision Points

Key information considered with the DPs included outcomes, clinical information (treatments and guidelines), and patient condition (measurements and comorbidities).

#### Outcomes

Two target outcome variables for T2DM were considered in this study, independently, to make the best use of the available data, as both HbA1c and fasting glucose are clinical measurements that can provide information about the T2DM state of the patient. The use of the 2 different outcome variables in separate scenarios enables a comparison between them and, in our perspective, offers the physician an opportunity to choose the one more relevant for a given clinical situation. In comparing the results for both scenarios, it is important to consider their different use in clinical practice. According to the guidelines, fasting glucose is used for diagnosis and primarily for making decisions about changing the dose of the prescribed medication. As the medication dose was not available from the data set, increasing the dose was considered to be a “no change” choice in this analysis. This probably explains that the percentage of “no change” cases for the glucose scenario was more than a factor of 1.2 higher than for the HbA1c scenario. According to the guidelines, only once the dosing is considered maximal, an uncontrolled fasting glucose value may be used to initiate the next progressive step in the treatment plan (ie, a different medication). Contrastingly, HbA1c, which reflects the patient’s glycemic status over the past 8 to 12 weeks, is specifically used to decide whether to initiate a treatment change to a different medication. From the data it appears that when a treatment change is based on fasting glucose, the status during follow-up is judged as “controlled” only approximately half as often as when the treatment change is based on HbA1c. This difference remains unexplained; however, the case might be that a fasting glucose cutoff of 7 mmol/L is a more stringent criterion than the HbA1c cutoff of 7%.

To mimic the situation in practice where measurements of HbA1c and fasting glucose are intermittently made in the same patient to follow disease status, we also tried to analyze a “mixed case” scenario in which both HbA1c and fasting glucose measurements were used to build the DPs (eg, use HbA1c to identify a noncontrolled situation, then use fasting glucose to evaluate the treatment follow-up). This approach, however, did not result in the expected increase in the number of DPs extracted from the data, so it was not pursued further. The observed discrepancy in results for the different target variables makes clear that for the implementation of the precision cohort analytics approach in practice in the future, a unified definition of when the disease is to be considered “controlled,” and a common decision on what metric is to be used to assess it, needs to be made.

#### Treatments and Guidelines

The data extraction retrieved all the usual medications used to treat T2DM, except for SGLT-2 inhibitors. Since 2021, the recommendations for high-risk patients include SGLT-2 inhibitors as medication. The absence of this treatment in the data set seemingly might indicate that the current Dutch guidelines for patients with T2DM were not followed in the study population. However, this can be explained not only by the fact that the data used here are from before 2021 but also by the fact that patients considered high risk are more likely to be referred to a specialist in secondary care, whereas the Nivel-PCD is concerned with primary care.

In the Netherlands, T2DM treatment over time has focused increasingly on lifestyle adjustments where possible, especially in the early stage of treatment where lifestyle improvement is the first treatment of choice except for high-risk cases [37]. This is, for example, reflected in a recent analysis of patients with T2DM diagnosed between 2015 and 2019, which showed that half of these patients did not receive antidiabetic medication prescriptions within 1 year of the diagnosis data [38]. Thus, lifestyle advices are probably often given; however, the fact that the “all_guidelines_variables_false” cohort was the fourth largest cohort for the HbA1c scenario seems to indicate that lifestyle adjustment is not integrally registered in the data fields within the Nivel-PCD (it is hardly imaginable that patients diagnosed with T2DM would receive no treatment at all). Incomplete registration of lifestyle treatment might have occurred because it is a default standard choice, or possibly because it is registered in the text fields of the patient dossiers that GPs use to make notes and are not collected in the Nivel-PCD. This will likely have led to an underestimation of the “healthy lifestyle advice” treatment group and likely to some misclassification, especially of patients in cohorts that involved “no treatment_stop” in this analysis.

Moreover, the merging of the nonpharmacologic treatments such as diet and exercise (registered as separate treatments in the Nivel-PCD) into a single “healthy lifestyle advice” as done in this study did not allow us to assess the individual contribution of each of the separate lifestyle interventions. However, this analysis still offers a way to evaluate the importance of lifestyle interventions for the patient’s health. Future studies could explore the efficacy of individual lifestyle adjustments as a first line of therapy for patients with T2DM, provided they are adequately registered.

The absence of medication dosage information in the available data set means that the current approach cannot be used to inform decisions regarding dosage changes. Similarly, the influence of medications that are not directly targeting diabetes, for example, the ones targeting blood pressure, was not considered in this retrospective analysis. Nevertheless, information on prescriptions of these other medications is available in the data set, and as drugs may have interactions, future studies might explore the possibility of including a larger spectrum of medications for a deeper analysis, leading to more refined models and decision-making tools.

#### Patient Condition

The idea to use both mobility and emotional states complementary to standard clinical measurements such as blood pressure, blood lipids, and creatinine came as a suggestion from a GP consulted for the study. We took care to also include the aspect that such impairment may be temporal instead of chronic, for example, in the case of a broken leg. As mentioned, for this exploratory analysis, we made subjective, intuitive choices to operationalize these conditions, which were not validated by independent experts. Therefore, they remain subject to debate. However, the fact that 11 (44%) of the 25 largest cohorts included “mobility impaired” suggests that it is indeed relevant to include a mobility assessment as a filter variable for defining the precision cohorts. The same holds for emotional state; however, with inclusion in only 3 (12%) of the 25 largest cohorts, the importance seems lower than for mobility impairment.

The patient condition characteristics used in this analysis are necessarily limited to the information available in the Nivel-PCD. Information about social behavior, ethnicity, socioeconomic status, medication adherence, and many other factors was not included, which might have a major impact on the disease outcome. This fact points to a world beyond what was analyzed in this study.

### Patient Similarity Modeling and Precision Cohort Construction

The selection of precision cohorts was done in a 2-step procedure, first a filtering step and then a similarity rating. The filtering step was based on guidelines, mobility, and mental state. The vast majority of the largest filter cohorts involved metformin and lifestyle advice as treatments, reflecting the importance of these as the first line of treatments according to the clinical guidelines. Patient similarity modeling and precision cohort construction were technically well feasible. However, data size limitations became apparent for the HbA1c scenario, where only 10 filter cohorts were considered sufficiently large (>200 DPs) to allow the construction of precision cohorts. This contrasts with the analysis of Ng et al [16] of the US EHR data, where the 75 largest T2DM cohorts based on filter variables had >200 DPs.

### Treatment Options and Outcomes Analysis

The technical feasibility of treatment options and outcome analysis was also well established. For the HbA1c target outcome, approximately half of the global treatment options resulted in better outcomes than the “no change” option. For fasting glucose as the target outcome, this was the case for only very few treatment options, suggesting that the use of fasting glucose as the target outcome needs careful consideration. The few examples of precision cohort–based treatment options and outcomes analysis clearly illustrated the lack of statistical power available with the current data set. This further underlines that the method has high data availability requirements. Because a correction for multiple testing has to be applied, reaching statistical significance for differences in outcomes of multiple treatment options proved challenging, even for filter cohorts with 1000 DPs, as demonstrated in Figures 3 and 5. It is estimated that to overcome this limitation, the data set size should be at least an order of magnitude larger.

### Future Perspectives

The method developed and applied for the Dutch primary care situation in this study aims to create precision cohorts that include a set of patients who are more similar to the patient of interest in different attributes, from classic clinical measurements to assessments of mobility state and even mental state. This would allow the physician to selectively consider a group of patients that were in a very similar situation to the patient of interest and view statistics on past outcomes of available treatment options. Over time, with the increase in the available patient information and developments in computational methods, the ability to thus incorporate past clinical experience to generate more personalized treatment options for individual patients is enhanced, thereby potentially contributing to better treatment outcomes.

In this study, we took a highly reductionist approach to defining treatment outcome, that is, an HbA1c or fasting glucose clinical test result that falls below a predetermined threshold. As such it is of a highly reductionist nature.

Although we recognize that a patient’s perspective and experience are crucial factors both in decision-making and in evaluating treatment efficacy, our data-driven approach was as yet unable to account for those aspects. Still, the technique can support shared decision-making by practitioner and patient because the information on expected treatment outcomes is more personalized toward the individual patient and therefore more relevant in the discussion when balancing risks and expected outcomes with patient preferences and values. Although this study did not explore the actual use of the precision cohort approach for shared decision-making in practice, it offers valuable insight into the potential use from a data availability perspective.

It is important to mention that this approach does not aim to replace or lessen the actions of the physicians but to provide refined tools to support them in the medical decision-making process.

The workflow elaborated in this study was applied to the T2DM case but can be applied to any other disease or health disorder for which rich data and guidelines are available. Indeed, Ng et al [16] applied their approach to hypertension and hyperlipidemia as well as T2DM. However, applying the approach to different diseases requires rerunning all the steps of the workflow to adapt for the different diseases, including patient selection, choice of target outcome variables, incorporation of applicable clinical guidelines, selection of salient features, and tuning of the similarity model.

In this study, data availability was identified to be a principally limiting factor for feasibility. Considering that further personalization will lead to yet smaller cohorts, it is evident that increasing the pool of data for the precision cohort approach is essential to achieve a more meaningful and more robust analysis. Given that approximately 1 million patients have T2DM in the Netherlands, there is a realistic perspective for this; however, it will require combining health data from different EHR sources nationwide, which is a challenge in itself. This problem is aggravated for diseases for which the amount of data (ie, patients) is much smaller or the spectrum of treatment choices is larger.

Although methods to reduce the presence of bias in the data were applied, having more data available offers a possibility to improve the workflow, especially with respect to better selection of the confounder variables. This may lead to a better generalization, improving the performance of the workflow as a whole.

### Conclusions

This study explored the feasibility of applying a patient similarity–based precision cohort approach to derive personalized treatment options for patients with T2DM treated in primary health care in the Netherlands using the Nivel-PCD. A previously published data analysis and modeling workflow for US EHR data was successfully adapted for this Dutch primary care setting, proving its potential for use in an LHS context. Although the approach proved technically well feasible, data size limitations need to be overcome before application for CDS purposes becomes realistically possible.

## Appendix

Multimedia Appendix 1 Features used for the similarity model for glycated hemoglobin. For each variable, the name, the use (filter or similarity), the type of feature (numerical, Boolean, etc), and the similarity weight (influence on the model) are shown.

Multimedia Appendix 2 Features used for the similarity model for fasting glucose. For each variable, the name, the use (filter or similarity), the type of feature (numerical, Boolean, etc), and the similarity weight (influence on the model) are shown.

## Abbreviations

CDS: clinical decision support
DM: diabetes mellitus
DP: decision point
EHR: electronic health record
GP: general practitioner
HbA1c: glycated hemoglobin
ICPC: International Classification of Primary Care
KNN: k-nearest neighbors
LASSO: least absolute shrinkage and selection operator
LHS: learning health system
LMNN: large margin nearest neighbor
MD: Mahalanobis distance
Nivel: The Netherlands Institute for Health Services Research
PCD: Primary Care Database
SGLT-2: sodium-glucose transport protein 2
T2DM: type 2 diabetes mellitus

## Data Availability

The data sets generated during and analyzed during this study are not publicly available. The Data from the Nivel Primary Care Database are available for research by other organizations upon approved data use request.
