# Supplementary figures and images for: Deriving Treatment Decision Support From Dutch Electronic Health Records by Exploring the Applicability of a Precision Cohort–Based Procedure for Patients With Type 2 Diabetes Mellitus: Precision Cohort Study
Source: Online J Public Health Inform. 2024 May 1;16:e51092. doi: 10.2196/51092 (PMC11097050; doi:10.2196/51092)

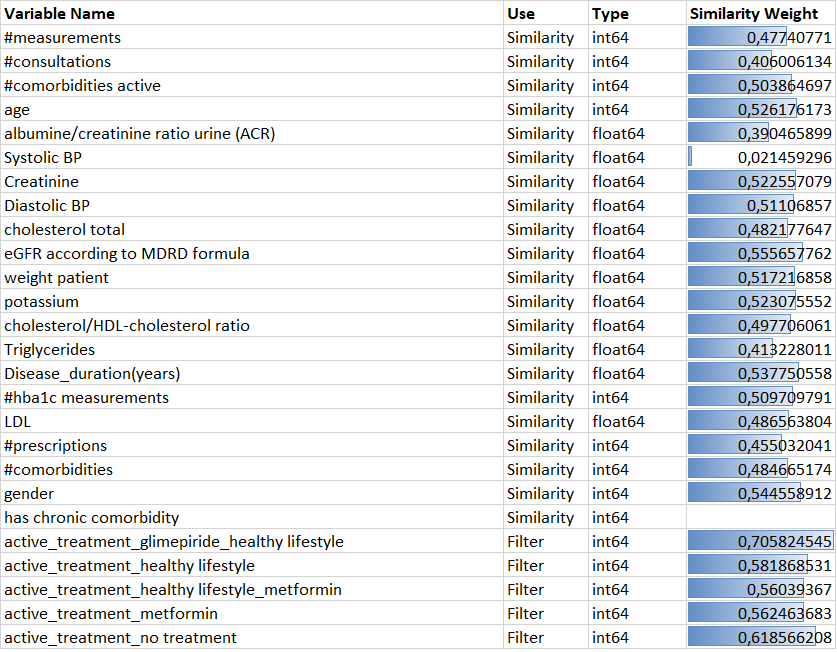

Supplement: Multimedia Appendix 1 [file ojphi_v16i1e51092_app1.png]

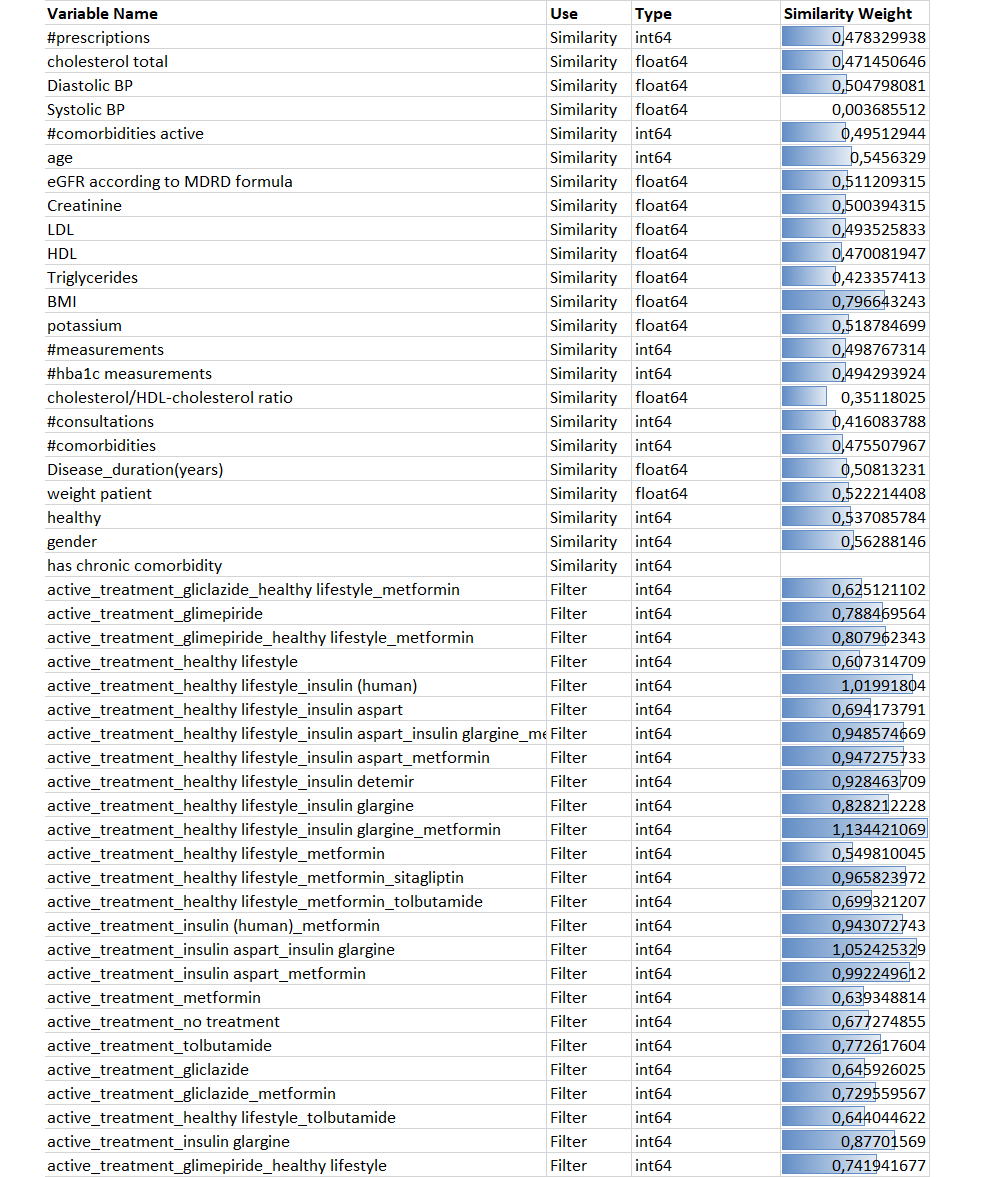

Supplement: Multimedia Appendix 2 [file ojphi_v16i1e51092_app2.png]
